# Oxidative Stress-Induced Cellular Senescence: Is Labile Iron the Connecting Link?

**DOI:** 10.3390/antiox12061250

**Published:** 2023-06-10

**Authors:** Lambros Nousis, Panagiotis Kanavaros, Alexandra Barbouti

**Affiliations:** 1 Department of Hygiene and Epidemiology, Faculty of Medicine, School of Health Sciences, University of Ioannina, 45110 Ioannina, Greece; lnousis@uoi.gr; 2 Department of Anatomy-Histology-Embryology, Faculty of Medicine, School of Health Sciences, University of Ioannina, 45110 Ioannina, Greece; pkanavar@uoi.gr

**Keywords:** oxidative stress, reactive oxygen species, labile iron, cellular senescence, telomeres, DNA-damage, lipofuscin, mitochondria, lysosomes

## Abstract

Cellular senescence, a cell state characterized by a generally irreversible cell cycle arrest, is implicated in various physiological processes and a wide range of age-related pathologies. Oxidative stress, a condition caused by an imbalance between the production and the elimination of reactive oxygen species (ROS) in cells and tissues, is a common driver of cellular senescence. ROS encompass free radicals and other molecules formed as byproducts of oxygen metabolism, which exhibit varying chemical reactivity. A prerequisite for the generation of strong oxidizing ROS that can damage macromolecules and impair cellular function is the availability of labile (redox-active) iron, which catalyzes the formation of highly reactive free radicals. Targeting labile iron has been proven an effective strategy to counteract the adverse effects of ROS, but evidence concerning cellular senescence is sparse. In the present review article, we discuss aspects of oxidative stress-induced cellular senescence, with special attention to the potential implication of labile iron.

## 1. Introduction

Cellular senescence, or simply senescence, is a cell state characterized by a generally irreversible growth arrest, driven by a variety of signals, including telomere shortening, oncogene activation, mitochondrial dysfunction, and reactive oxygen species (ROS) [1,2,3,4]. Senescent cells display higher levels of macromolecular damage, altered metabolism, and a specific secretory phenotype [1,2,3,4]. This fundamental biological process has several beneficial functions for the organism, as it prevents the propagation of unwanted cells, and triggers their clearance by the immune system [2,5]. However, excessive accumulation of senescent cells within tissues and organs contributes to tissue dysfunction, inflammation, and tumorigenesis in aged organisms [2,3,6]. Therefore, unveiling the underlying mechanisms that determine senescence initiation and establishment is of the utmost clinical importance.

ROS are common drivers of cellular senescence. Excess ROS can oxidize all essential macromolecules (DNA, lipids, proteins) and impair lysosomal and mitochondrial function [7,8,9]. These effects are common features of cellular senescence. It has to be emphasized, however, that ROS is an umbrella term for a group of molecules with varying chemical reactivity: some are strong oxidizing agents, and some are not [10]. A requirement for the generation of extremely damaging ROS is the availability of labile (redox-active) iron, which catalyzes the conversion of relatively weak oxidants to highly reactive free radicals [11]. Thus, the diminution of labile iron may reduce their generation and their subsequent adverse effects. In the present review article, we focus our interest on the underlying mechanisms that contribute to ROS-induced cellular senescence. Special attention is given to the potential role of labile iron in this process. 

## 2. Oxidative Stress

### 2.1. Oxygen: A Double-Edged Sword for Aerobes

Molecular oxygen (O_2_) makes up approximately 21% of the air in Earth’s atmosphere. Its appearance roughly 2.4 billion years ago allowed the emergence and expansion of complex eukaryotic life. In aerobic cells, O_2_ is reduced to water in electron transport chains (ETC), producing large amounts of energy. In animal eukaryotic cells, ETC is located in mitochondria and the electrons for O_2_ reduction derive from catabolic reactions. 

Although O_2_ is indispensable for aerobic beings, its unavoidable metabolic byproducts can react with various substances within the cells such as DNA, proteins, lipids, and carbohydrates, and oxidize them [10]. In such a hostile environment, aerobes survive only because during their long evolution they have developed efficient reducing defenses [12]. The continuous generation of these O_2_ metabolites—widely known as ROS—is counterbalanced by their elimination [10]. However, under certain circumstances, this equilibrium can be shifted in favor of the oxidants, disrupting cellular and, by extension, organismal, homeostasis. This state, defined as oxidative stress, has been implicated in various human diseases [10]. 

### 2.2. ROS Generation and Regulation

#### 2.2.1. ROS Generation

**Figure 1:**
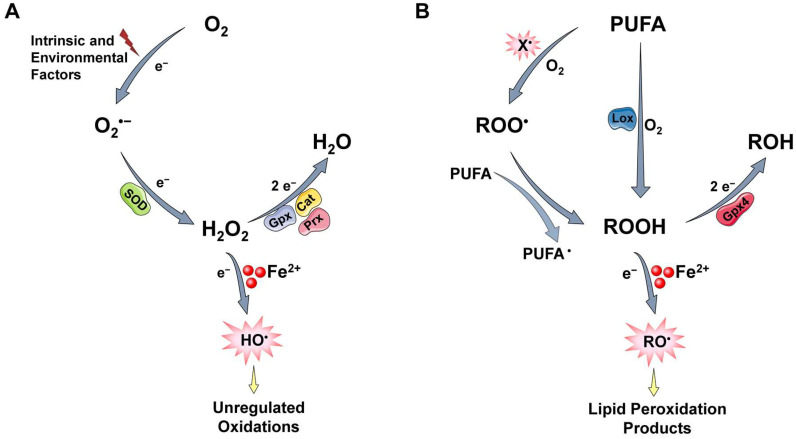
Generation of ROS and lipid peroxidation products. (**A**) The majority of O_2_ consumed in eukaryotic cells is reduced safely to water (H_2_O) by the enzyme cytochrome c oxidase in the respiratory ETC, without causing collateral damage. However, a small amount of O_2_, even under normal conditions, undergoes partial reduction to produce superoxide (O_2_**^•−^**), which is usually the first ROS to be formed. O_2_**^•−^** is rapidly converted to hydrogen peroxide (H_2_O_2_) by superoxide dismutase (SOD), while the generated H_2_O_2_ is further converted to H_2_O through the action of catalases (Cat), glutathione peroxidases (GPx), and peroxiredoxins (Prx). Alternatively, in the presence of available ferrous iron ions (Fe^2+^), H_2_O_2_ is reduced non-enzymatically by one electron, producing the highly reactive HO**^•^**. O_2_**^•−^** and H_2_O_2_ are moderately reactive and can interact with a limited number of cellular macromolecules. On the contrary, HO**^•^** is highly reactive and it can oxidize indiscriminately, with high rate constants, most, if not all molecules in living cells. (**B**) Lipid-derived reactive species are generated through lipid peroxidation when reactive species attack lipids and especially polyunsaturated fatty acids (PUFA). The process begins with the incorporation of O_2_ and the generation of lipid hydroperoxides (ROOH). Oxidation of PUFA to ROOH can proceed either enzymatically by lipoxygenase (Lox) or non-enzymatically when a PUFA is oxidized from a free radical (X**^•^**) to a lipid peroxyl radical (ROO**^•^**) which subsequently attacks an adjacent PUFA. ROOH are relatively unstable and can be reduced to their corresponding innocuous alcohols (ROH) via the glutathione peroxidase 4 (GPx4). Yet, when ROOH levels elevate and Fe^2+^ is available, extremely potent alkoxyl radicals (RO**^•^**) are generated, capable of oxidizing new PUFA, and producing damaging end-products of peroxidation. The figure was partly generated using Servier Medical Art, provided by Servier, licensed under a Creative Commons Attribution 3.0 unported license.

The majority of O_2_ consumed in eukaryotic cells is utilized in mitochondria to produce chemical energy. Complex IV (also known as cytochrome c oxidase), the last e nzyme in the respiratory ETC, transfers four electrons from cytochrome c to O_2_. This concerted tetravalent reduction converts O_2_ to water without the formation of reducing intermediates that could cause collateral damage and generates a proton gradient in the inner mitochondrial membrane that is used to drive ATP synthesis. However, a small amount of O_2_, even under normal conditions, undergoes partial reduction to produce ROS such as the superoxide anion radical (O_2_**^•−^**), which is usually the first ROS to be formed, as well as hydrogen peroxide (H_2_O_2_), and the hydroxyl radical (HO**^•^**) (Figure 1A). These byproducts of the normal metabolism of O_2_ exhibit different chemical reactivity. Particularly, O_2_**^•−^** and H_2_O_2_, which represent the one- and two-electron reduction products of O_2_, respectively, are moderately reactive and can interact with a limited number of cellular molecules [10]. On the contrary, HO**^•^** is regarded as the most reactive oxidant produced in vivo and it can oxidize indiscriminately, with high rate constants, most, if not all molecules in living cells [11]. HO**^•^** is mainly produced in biological systems by the reaction of H_2_O_2_ with ferrous iron ions (Fe^2+^) through the Fenton reaction (see Section 2.3) [11,13,14]. 

The generation of the aforementioned intermediates of incomplete O_2_ reduction within cells can be facilitated by various intrinsic factors [10]. Mitochondria are considered a major intracellular source of ROS [15,16]. Electrons can leak from ETC (mainly complex I and complex III) and generate O_2_**^•−^**, which can subsequently produce other downstream ROS [17]. Under normal conditions, mitochondria produce low amounts of ROS; however, dysfunctional mitochondrial are often associated with excessive ROS generation. 

NADPH oxidase 2 (Nox2) is another major source of cellular ROS. This membrane-bound enzyme complex is found in phagocytes, cells that constitute our first line of defense against invading pathogens [18]. When activated, phagocytes exhibit a marked increase in O_2_ uptake called the respiratory burst. Nox2 reduces O_2_ and releases high amounts of O_2_**^•−^**. Superoxide dismutases (SOD) can convert O_2_**^•−^** into H_2_O_2_, which is further converted to hypochlorous acid (HOCl) in a reaction catalyzed by myeloperoxidase (MPO). While these reactive species are essential for effective antimicrobial defense, they unavoidably cause collateral damage to neighboring cells.

Except for Nox2, other members of the NOX family occupy different cellular localizations and generate low amounts of ROS associated mainly with cell signaling [19]. ROS are also generated by a variety of other oxidases prominently present in different cellular compartments [10]. Reduced oxygen intermediates can be also derived from interactions with environmental factors called the “exposome”, which include drugs, toxicants, pollutants, nutrients, physical stressors (e.g., ionizing radiation), and psychological stressors (lifestyle) [10,20]. 

Reactive species are also generated through lipid peroxidation, a process under which highly reactive radicals such as HO**^•^** attack lipids, especially polyunsaturated fatty acid (PUFA) side chains [21]. The process begins with the incorporation of O_2_ and the generation of lipid hydroperoxides (ROOH), a reaction that can proceed either enzymatically or non-enzymatically (Figure 1B). In the first case, PUFA serves as a substrate for lipoxygenase enzyme (LOX), which forms hydroperoxyl groups (ROOH) at the carbon position of allylic chains. In the second case, a reactive free radical, such as HO**^•^**, which is mainly generated through Fenton-type reactions, abstracts a hydrogen atom from a methylene (-CH_2_-) group, forming a carbon radical. These reactions are favored in bis-allylic positions; that means, the more double bonds, the more oxidizable the PUFA. The most likely fate of a carbon radical is to react with O_2_, generating a peroxyl radical (ROO**^•^**). The latter, if not scavenged, attacks an adjacent fatty acid side chain, generating a new carbon radical, which can, in turn, react with O_2_,propagating lipid peroxidation chain reactions, while ROO**^•^** itself abstracts a hydrogen atom to form a lipid hydroperoxide (ROOH). ROOH are not highly reactive and can be safely reduced to their corresponding innocuous alcohols via glutathione peroxidase 4 (GPx4), an enzyme that acts on peroxidized fatty acids located within membranes and lipoproteins. Yet, like H_2_O_2_, if ROOH levels elevate and Fe^2+^ is available, highly reactive alkoxyl radicals (RO**^•^**) are generated. The latter are extremely potent species, capable of oxidizing new PUFA, amplifying the lipid peroxidation process. The continued production of lipid ROO**^•^** and RO**^•^**, and their decomposition to produce end-products of peroxidation (such as isoprostanes, malondialdehyde, and 4-hydroxy-2-nonenal) have detrimental effects on cellular membranes and play a critical role is cell death pathways including apoptosis and ferroptosis [22,23,24]. 

#### 2.2.2. Protective Mechanisms

It is obvious from the above that aerobes live in an extremely unfriendly oxidative environment and survive only because they contain efficient reducing systems. The main cellular defense against oxidants is a network of enzymes that catalytically reduce relatively weak O_2_-derived intermediates to less reactive or innocuous molecules [25]. Particularly, SOD is highly efficient in reducing O_2_**^•−^** to H_2_O_2_, which is converted to water through the action of catalases (Cat), glutathione peroxidases (GPx), and peroxiredoxins (Prx) (Figure 1A). Moreover, small antioxidant agents synthesized by aerobes, such as the tripeptide glutathione (GSH) which serves as a substrate for GPx, can be oxidized by reactive species, protecting, in this manner, more important cellular biomolecules [10]. However, some reactive species, such as the HO**^•^** and RO**^•^,** are very strong oxidizing agents. Once generated, they react very quickly with almost any molecule that happens to be in their immediate vicinity and oxidize it. It follows that it is unlikely to be eliminated by endogenous or exogenous scavengers (antioxidants) garnered from the diet [26]. On the other hand, enzymatic defense systems or agents that control the formation of such reactive species seem to be much more effective. Reducing enzymes, as mentioned above, act in concert to prevent the generation of reactive free radicals such as HO**^∙^** and RO**^∙^** (Figure 1A,B). The sequestration of metal ions, particularly iron ions, is also of major importance for preventing their generation. For instance, proteins such as transferrin or ferritin that bind iron in the circulation or within cells, respectively, keep this metal ion in a redox-inactive state. In this way, they minimize their ability to participate in reactions that generate damaging free radicals (see Section 2.3) [11,14]. Notably, diet—and especially the Mediterranean type—contains a plethora of bioactive compounds with iron-chelating capacity, which, when they reach the cell interior, protect cells against oxidative stress [26,27,28,29,30,31,32]. 

### 2.3. Labile Iron and Its Key Role in ROS-Induced Toxicity

Iron is an essential trace element for almost all living cells and organisms. It is utilized as a cofactor of numerous proteins and enzymes that support important biochemical functions, including O_2_ transfer, DNA repair, and energy production [11,14,33,34]. Although vital, iron is at the same time a potential pro-oxidant, as it serves as the major catalyst for the generation of reactive free radicals through Fenton-type reactions. During Fenton-type reactions, peroxides, relatively weak oxidants, convert to extremely potent free radicals such as HO**^•^** and RO**^•^** (Reactions (1) and (2)).

Fe^2+^ + H_2_O_2_ → Fe^3+^ + HO**^•^** (1)

Fe^2+^ + ROOH → Fe^3+^ + RO**^•^** (2)

For this reason, mammals are equipped with sophisticated mechanisms which tightly regulate iron homeostasis, so they can fulfill their metabolic needs for iron and minimize its toxic effects [11,14]. Thus, the hepatic peptide hormone hepcidin coordinately controls systemic iron levels, while iron-regulatory proteins 1 and 2 (IRP1 and IRP2) regulate cellular iron levels [11,34]. Moreover, most of the body’s iron is kept in a redox-inert state: circulating iron is tightly bound to the iron carrier transferrin, while most of the intracellular iron is well protected in the active sites of enzymes, or safely stored within cytosolic ferritin [11,33].

However, a small fraction of the total intracellular iron is accessible to peroxides and contributes to the generation of extremely reactive free radicals via Fenton-type reactions [35,36]. This fraction is known as labile iron, and its molecular nature is one of the most obscure facets in iron biology. Due to its dynamic nature, the determination and precise quantification of labile iron has been an obstacle, and for a long time there was a controversy about the existence of a pool of free intracellular iron. Although hard to define, labile iron is usually defined as the iron that: (i) is redox-active, (ii) can be sequestrated by weak iron chelators, and (iii) has a transitory nature, as it is destined for storage, export, or metabolic utilization [36]. 

Free radicals generated via Fenton-type reactions are highly reactive and short-lived; thus, they attack and oxidize chemical groups in the vicinity of their formation. Hence, iron-binding sites on macromolecules are particularly sensitive to oxidation and serve as centers for Fenton-type reactions [37]. Therefore, sites that are prone to oxidation can fluctuate according to the levels of labile iron: when labile iron increases, more sites may become susceptible to oxidation; on the contrary, when it decreases, iron moves away from these sites, making them resistant to oxidation. 

### 2.4. The Pleiotropic Effects of ROS on Normal Proliferating Cells

**Figure 2:**
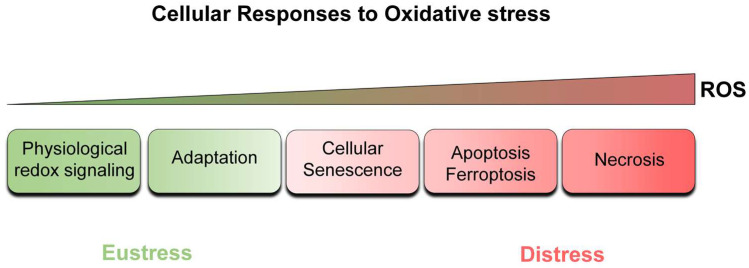
Cellular responses to oxidative stress. Free radicals and other reactive intermediates formed as by-products of cellular aerobic metabolism are continuously generated by endogenous or exogenous sources and eliminated by reducing defense systems. At low steady-state levels, by oxidizing specific targets, ROS regulate signaling pathways implicated in various physiological processes. It follows that defense systems coordinately attenuate ROS to minimize oxidative damage, whilst permitting enough to remain to fulfill useful roles (eustress). However, deviation from this steady state leads to unspecific oxidation, disrupting redox signaling and/or causing macromolecular damage (distress). Depending on the severity of the damage, cell responses can range from permanent growth arrest (cellular senescence) to programmed cell death (apoptosis or ferroptosis), or in more extreme conditions, necrotic cell death. Adaptive responses that modulate ROS levels and counteract damaging effects can also be elicited.

Cellular responses to ROS depend on the nature, the level, and the duration of the stimulus, as well as the cells’ ability to cope with the oxidants to which they are exposed (Figure 2). To survive in such oxidative environments, cells have evolved sophisticated mechanisms to regulate ROS. Yet, although extremely important, these mechanisms cannot eradicate them. However, as it turns out, this is not a flawed outcome of the evolutionary process. Basic steady-state levels of ROS, and especially H_2_O_2_ via reversible oxidation of specific protein targets, can regulate signaling pathways (redox signaling) implicated in various physiological processes, such as cell proliferation, differentiation, migration, and angiogenesis [10]. The maintenance of a state of low ROS levels which plays useful roles is defined as “oxidative eustress”, from the Greek word *eu*, which means “good” [10,38]. In line with this essential role of ROS as physiological signaling agents, cells usually respond to moderately elevated ROS levels by improving reducing defenses, which help cells to defend against the insult and survive in the new environment (adaptation or hormesis) [10,12]. 

However, elevated levels of ROS can cause damage to all essential macromolecules, impairing cellular function and leading to various pathological conditions. This state is referred to as “oxidative distress” [10,38]. Excessive amounts of ROS trigger cell death by regulated pathways (e.g., apoptosis, ferroptosis) or, in more extreme conditions, necrotic cell death. Senescence, a permanent arrest of cell proliferation, can be also triggered by elevated ROS. Senescence appears to lie between adaptation and cell death, as it is often associated with survival but also with permanent structural and functional changes. Subsequent sections describe the mechanistic aspects of oxidative stress-induced cellular senescence with an attempt to unveil the putative role of labile iron in this cellular process.

## 3. Cellular Senescence

### 3.1. A Brief Historical Overview and Some General Aspects of Cellular Senescence

Cellular senescence is a fundamental biological process, mainly characterized by a prolonged and generally irreversible arrest of cell proliferation. It was originally described six decades ago when Leonard Hayflick and Paul Sidney Moorhead showed in their seminal paper that normal diploid cells isolated from human fetuses gradually lost their ability to divide in vitro, despite the presence of ample space, growth factors, and nutrients in the culture medium [39]. Notably, the non-dividing cells remained alive and metabolically active over a long period until the eventual degeneration of the culture. This phenomenon was termed senescence, from the Latin word senex, which means “growing old”, as the authors assumed that it could play a causal role in organismal ageing. The underlying molecular explanation came a few decades later with the discovery that permanent cell cycle arrest is the result of telomere attrition [40]. Telomeres—from the Greek words *telos* (end) and *meros* (part) [41]—are the terminal chromatin structures that mask the natural ends of eukaryotic chromosomes and protect them from degradation and fusion [42,43]. However, the DNA at the very end of a linear chromosome (that is, the telomere) cannot be fully copied during replication. This phenomenon, commonly referred to as the “end-replication problem”, was observed in 1972 by James Watson [44]. At nearly the same time, Alexei Olovnikov first postulated that due to their incomplete replication, telomeres progressively becoming shorter with each cell doubling, and when this shortening reaches some threshold value, cells stop dividing and senesce [45]. For a long time, there was a controversy about whether telomere shortening and senescence had any relevance to organismal ageing or whether they were just a tissue culture phenomenon. This changed abruptly during the past few decades, as several studies in humans have shown that telomeres shorten with age, and shortening is associated with increased mortality risk [43,46,47,48]. Similarly, research in animal models revealed that short telomeres led to ageing-associated degenerative defects and loss of organismal viability [49,50,51], while telomere elongation delayed normal ageing [52]. 

Moreover, it became clear that apart from telomere attrition, several diverse signals can elicit senescence phenotypes. For instance, various stressors including oxidants, genotoxic agents, hypoxia, and aberrant activation of oncogenes trigger premature senescence, usually in the absence of telomere shortening [1,2,53]. Besides, the role of senescence has recently expanded beyond the contexts of telomere attrition or stressful insults, as cells with senescent features have been identified during embryogenesis, to particular transient anatomical structures, and during specific time windows of development in evolutionary distant organisms ranging from mammals to fish [2,54]. 

Conceivably, senescence as a stable growth-arrest program has evolved as a mechanism to prevent the propagation of unwanted cells and also to trigger their clearance by the immune system [2,5]. During development, senescence regulates embryonic growth and patterning [2,54], while in adulthood, senescence counteracts the uncontrolled growth of damaged cells and is a crucial barrier against cancer progression. Within this framework, senescence represents a beneficial response, essential for maintaining tissue homeostasis. Yet, senescent cells are not inert; they remain alive for prolonged periods and release factors that can harm neighboring healthy cells and the very cells that produce these factors. Accordingly, the accumulation of senescent cells within tissues and organs—when the immune system fails to efficiently remove them or when senescence persists—contributes to tissue dysfunction and gives rise to pathological manifestations, organ ageing, and age-related diseases [2,3,6,55,56]. Comprehensively, senescence is implicated in several physiological functions, but also in a wide variety of age-related pathologies, and displays beneficial effects as well as detrimental consequences on organismal health, depending on the context. 

### 3.2. Features of Senescent Cells 

**Figure 3:**
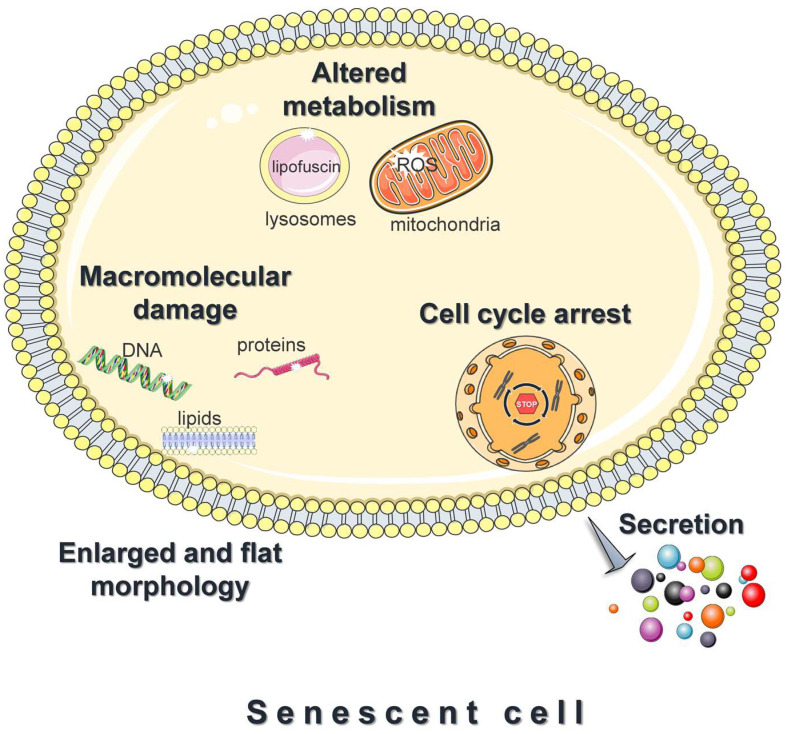
Features of senescent cells. Cellular senescence is the essentially irreversible growth arrest that occurs when cells experience stressful insults or certain physiological processes. Common features of senescent cells are an abnormally enlarged and flat morphology, altered metabolism, and the accelerated accumulation of damage to DNA, proteins, and lipids. Moreover, senescent cells adopt a hyper-secretory phenotype, known as the senescence-associated secretory phenotype (SASP). The figure was partly generated using Servier Medical Art, provided by Servier, licensed under a Creative Commons Attribution 3.0 unported license.

A prominent feature of senescent cells is the irreversible growth arrest (Figure 3), which is largely mediated through either one or both the p53/p21 and the p16^Ink4a^/pRB signaling pathways [1,57]. Both pathways involve multiple upstream regulators and downstream effectors, and cross-regulate each other [58]. In addition, to some extent, they respond to different stimuli; DNA-damaging signals such as oxidants, and oncogenic or genotoxic stress, largely trigger senescence via the p53/p21 axis [57]. Upon entering senescence, cells display an abnormally enlarged and flat morphology with an increase in the cytoplasm-to-nucleus ratio. Moreover, they exhibit deregulated metabolism and accelerated accumulation of damage to DNA, proteins, and lipids (Figure 3) [59,60,61]. Last, but not least, senescent cells adopt a hyper-secretory phenotype, known as the senescence-associated secretory phenotype (SASP) (Figure 3) [1,57]. The exact composition remains elusive and varies significantly [62]; however, the SASP mainly includes proinflammatory cytokines, chemokines, angiogenic factors, matrix metalloproteinases, and ROS. Although SASP facilitates tissue homeostasis, when chronic, it mediates the pathophysiological effects of senescence; by acting in an autocrine or paracrine mode, the secreted factors reinforce and spread senescence, promote persistent chronic inflammation, stimulate tumorigenesis, impair the function of stem and progenitor cells, etc. [1,57]. This explains why senescent cells, although present in relatively low numbers in vivo, have such devastating consequences. 

## 4. Intracellular Damage in Oxidative Stress-Induced Cellular Senescence: Is Iron Involved?

A well-known feature of cellular senescence is its intimate association with macromolecular damage: various damaging insults signal cellular senescence, while the accelerated accumulation of macromolecular damage is an almost universal hallmark of senescent cells [1]. The senescent state is also characterized by an altered metabolic profile. Lysosomes exhibit progressively deteriorated function and accumulate lipofuscin, a non-degradable aggregate of intracellular catabolism [5]. Lysosomal dysfunction attenuates mitophagy (a mitochondrial quality control mechanism that degrades damaged mitochondria) leading to the accumulation of damaged mitochondria which often produce elevated ROS levels [63]. This, in turn, targets lysosomes and enhances macromolecular damage, aggravating senescence phenotypes [63]. 

ROS are common drivers of cellular senescence and trigger all the aforementioned senescence traits. As analyzed in the above sections, the term ROS encompasses species with varying chemical reactivity. For instance, O_2_^•−^ and H_2_O_2_ are not reactive enough to directly oxidize cellular macromolecules and induce damage. However, in the presence of labile iron, peroxides can be converted to extremely reactive free radicals such as HO^•^ and RO^•^ (Figure 1, Reactions (1) and (2)). These free radicals are short-lived and highly reactive; thus, they attack and oxidize their targets close to the site of their formation (diffusion-controlled reactivity). Consequently, when peroxides are elevated (oxidative stress conditions), labile iron can trigger single- or double-strand breakage and oxidative modifications to DNA bases, oxidative damage to proteins, and peroxidation of lipids [7]; all these effects can elicit cellular senescence. Moreover, labile iron is not uniformly distributed within cellular compartments. Lysosomes and mitochondria, organelles that undergo the most remarkable alterations during senescence, contain higher amounts of labile iron compared with other cellular compartments [64,65]. The high iron content makes these organelles extra sensitive in oxidative stress conditions [8,9,66,67]. Considering the above, the implication of labile iron in the mechanisms of oxidative stress-induced cellular senescence is a quite reasonable assumption. In the subsequent sections, we discuss the potential interplay of labile iron, oxidative stress, and senescence and examine curevidence for the role of labile iron in this cellular process. 

### 4.1. DNA Damage

#### 4.1.1. Activation of the DNA Damage Response Pathway

Nuclear DNA, although bearing hereditary information, is particularly susceptible to oxidative stress. ROS cause various lesions to DNA, such as single- and double-strand breakage, and oxidation to purines and pyrimidines [68]. Eukaryotic cells are equipped with a complex network known as the DNA damage response (DDR), which protects the genome from detrimental insults [69]. The DDR machinery can detect DNA lesions and set the cell fate depending on the cell type and the severity of the damage [70,71,72]. When genotoxic agents such as ROS trigger DNA damage, the DDR machinery temporarily arrests cell proliferation and gives time for efficient DNA repair. After the damage is repaired, the cell exits from the arrest phase and resumes cell cycle progression. However, if the damage is severe and irreparable, the prolonged DDR activation may cause a permanent cell cycle arrest and the senescence phenotype, or otherwise may initiate cell death programs (apoptosis) [70,71,72].

The DDR initiates with the recognition of the DNA damage by sensor proteins and the activation of signaling protein kinases such as ataxia-telangiectasia-mutated (ATM) and ATM- and Rad3-related (ATR) kinases. ATM and ATR phosphorylate histone γ-H2A.X, which regulates various downstream mediators (such as 53BP1) to coordinate the DDR machinery, and the downstream kinases CHK1 and CHK2, which ultimately transmit the damaging signal to p53. The p53 transcription factor regulates the expression of the cyclin-dependent kinase (CDK) inhibitor p21, leading to cell growth arrest and senescence [73]. 

Telomere shortening due to cellular replication also results in DDR activation and induction of cellular senescence [70,71]. Interestingly, as it turns out, oxidative stress interferes with telomere homeostasis, and ROS-induced telomere dysfunction may signal persistent DDR activation and senescence, which can be either dependent on or independent of telomere length [43,74,75,76].

#### 4.1.2. Telomeres and how they signal senescence

Telomeres are specialized structures at the ends of eukaryotic chromosomes essential for maintaining genomic stability. They consist of tandem nucleotide repeats coated with a six-member protein complex known as the shelterin [43]. Human telomeres consist of roughly 5–15 kilobases that terminate in a 3′ single-stranded overhang of approximately 50–200 nucleotides. In humans and mammals, their basic DNA repeat is the hexanucleotide sequence 5’-TTAGGG-3’ in the strand that contains the 3’-end [77]. The telomeric end folds back onto itself, forming a lariat-like structure (t-loop), while the single-stranded overhang invades the telomeric double-stranded region [78]. This highly organized structure, which is arranged and stabilized via the shelterin proteins, safeguards the single-stranded terminus from being recognized as a potential breakpoint and the resulting erroneous activation of the DNA damage response (DDR) machinery [79]. 

Telomeres have been intimately linked with the onset of senescence [43]. Their length decreases with each cellular replication in almost all somatic cells of adult organisms, due to an inability of the replicative polymerases to complete the synthesis at the ends of linear chromosomes. This phenomenon can be counteracted by telomerase, the specialized enzyme that compensates for telomere loss by elongating chromosomal ends. However, since telomerase is repressed in the majority of human somatic cells, telomeres become progressively shorter as cells divide, and when tthey get below a crucial length, they cannot sufficiently bind the shelterin and the telomeric loop destabilizes [74]. This results in the exposure of their terminus region, which is sensed as a double-strand break and accumulates proteins involved in DDR machinery. The DDR signaling through the induction of cell cycle inhibitors promotes the stable arrest of the cell cycle, also known as replicative senescence [70,80]. 

#### 4.1.3. New Insights into Oxidative Stress-Induced Telomere Shortening and/or Dysfunction

The initial hypothesis concerning the link between telomeres and senescence was that the former are merely a “biological clock” that measures mitotic time and stops proliferation after a more or less fixed number of cell divisions (when telomere length reaches a certain cutoff point) [43]. This simplistic concept has been switched to a more complicated perception, as it has become apparent that telomere homeostasis strongly correlates with oxidative stress, and it was proposed that oxidative stress may contribute to telomere shortening more than the end-replication problem alone [81,82]. During the past few decades, several in vitro studies in human cultured cells have revealed that oxidative stress accelerates telomere shortening and inhibits cell proliferation [76,81,83,84,85], while treatment with antioxidants prevents oxidative stress-induced telomere shortening and extends the proliferative lifespan [81,86]. Moreover, evidence from animal models and human studies supports the negative correlation between oxidative stress and telomere length [87]. Remarkably, telomere loss is not solely a stochastic event, since telomeric DNA is more vulnerable to oxidative damage compared with the bulk of the genome, and this is due to many reasons.

First of all, the damage that occurs within telomeric regions is less efficiently repaired compared with elsewhere in the chromosome [71,88,89]. This is mainly because the shelterin protein-complex coats telomeres, rendering their damage unrecognizable by the DNA repair enzymes [71,88,90,91]. The unrepaired, and therefore persistent, telomeric damage causes a prolonged DDR signaling, ultimately leading to permanent cell cycle arrest and the senescence phenotype [71,88,92]. 

Apart from being hard to repair, telomeres are also more sensitive to oxidative stress compared with the bulk of the genome, a feature recently called “TelOxidation” [93]. One main reason for telomeres’ susceptibility to oxidative stress is their high content in guanine, the DNA base most vulnerable to oxidation. Ιn vitro studies have shown that under oxidative stress conditions, 8-oxo deoxyguanine (8-oxoG)—the primary product of deoxyguanine oxidation—is more predominant in telomeric sequences compared with non-telomeric sequences [85,94]. A recent study in immortalized cells by Fouquerel et al. has shown that the persistent 8-oxoG induction exclusively within telomeric regions hastened telomere shortening, impaired cell growth, and triggered replication stress, even though telomeres are only a minor proportion of the chromosomes [95]. The authors proposed that the persistent telomeric 8-oxoG induction interferes with telomere replication, aggravating their shortening. 

While telomeric damage can occur through the direct attack of oxidants on DNA, it may also arise by oxidative modifications of the cellular nucleotide pool [96]. In this case, the insertion of oxidized nucleotides—particularly the oxidized form of the guanine nucleotide—inhibits telomerase, preventing further telomere extension [77,97,98,99]. In this way, the oxidation of free nucleotides hastens telomere shortening and loss. 

An additional threat for telomeres under oxidative stress conditions is the dissociation of shelterin proteins. 8-oxoG lesions or intermediates of base excision repair in telomeric DNA reportedly disrupt the binding of the TRF1 and TRF2 (telomere repeat binding factors 1 and 2) proteins, which are essential for telomere stability [100]. 

Notably, although the majority of studies so far supports that oxidative stress hastens telomere shortening and the onset of senescence, recent reports suggest otherwise. Barnes et al., have shown that in non-diseased cells, telomeric 8-oxoG formation induced multiple markers of p53-dependent premature senescence, but remarkably, this was not accompanied by telomere shortening. Yet, their results suggest that telomeric 8-oxoG lesions in non-diseased cells activate DDR signaling and enforce p53-dependent premature senescence by disrupting DNA replication and increasing telomere fragility [75]. Moreover, in vivo experiments confirmed that infiltrating neutrophils in the liver induces ROS-mediated telomere dysfunction and senescence in neighboring hepatocytes, which was not accompanied by telomere shortening [76]. 

#### 4.1.4. The Putative Role of Labile Iron in Oxidative Stress-Mediated DNA Damage and Cellular Senescence

ROS accumulation can damage DNA and activate the DDR pathways, causing various responses, including cellular senescence. Evidence in the literature is clear in highlighting that the intracellular availability of labile iron represents a prerequisite for ROS-induced adverse effects [11,14,101]. This is because iron catalyzes the conversion of relatively unreactive peroxides to extremely reactive free radicals that can instantly attack and oxidize all essential cellular macromolecules, impairing cellular function (see Figure 1 and Reactions (1) and (2)). Previous in vitro and in vivo studies from our research team revealed that the diminution of intracellular iron protects against oxidative stress-induced DNA damage and cell death [101,102,103,104,105]. Given that iron is essential for the generation of extremely reactive free radicals that can damage DNA (telomeric or non-telomeric), it may well contribute to oxidative stress-mediated cellular senescence. Recent studies have indicated that excess intracellular iron accelerates cellular senescence by damaging the DNA via Fenton chemistry [106]. This process was defined by Sfera et al. as “ferrosenescence” [106]. Moreover, iron levels have been associated with specular changes in p53 activity in mouse hepatocytes and rat liver [107]. 

### 4.2. Protein Oxidation

Apart from DNA damage, impaired proteostasis is another prominent feature of senescent cells [108]. A well-known cause of proteotoxicity is ROS, which provoke oxidative modifications leading to protein misfolding and aggregation. ROS may directly attack and cleave protein backbones, generating protein fragments, or may oxidize protein amino acids, especially the aromatic and sulfur-containing ones [109]. Oxidative modifications may also be induced via indirect reactions with reactive intermediates originating from lipid or carbohydrate oxidation [109,110]. 

Notably, some types of protein oxidation—for example, certain oxidations of thiol groups—can be reversed enzymatically. Such oxidation may regulate signaling mechanisms and are relevant in physiological processes (this is referred to as redox signaling) [11,60,111]. Nevertheless, most types of oxidative damage in proteins cannot be reversed and, since their accumulation compromises cell function, their elimination is extremely important. The maintenance of proteostasis is attained through precisely coordinated networks that rapidly reverse or degrade oxidized proteins [60]. To some extent, non-reversibly oxidized proteins are degraded by the proteasomal or autophagy–lysosomal systems. However, when the rate of their formation overwhelms the rate of their degradation, oxidized proteins accumulate, raising the risk of the formation of insoluble protein aggregates which may further impair the activity of proteasomal and lysosomal degradation systems [112]. 

Oxidized proteins are elevated in oxidative stress-related pathologies, aged organisms, and senescent cells as well [7,60,108,109,113,114,115]. Although not necessarily indicative of senescent cells, the accumulation of oxidized proteins and protein aggregates is tightly related to senescence [7,115]. In agreement with that, protein degradation by proteasomal and lysosomal systems is affected in senescent cells [7]. Moreover, oxidized proteins form lipofuscin, a non-degradable aggregate that represents a universal hallmark of senescence cells, as will be discussed below. 

Although the interactions of labile iron with cellular proteins are ill-defined, there is evidence that some ROS-mediated signaling pathways are dependent on iron availability [11]. 

### 4.3. Lipid Oxidation 

A large number of existing studies support that senescent cells alter lipid metabolism, although our knowledge concerning the specific lipid metabolite composition or its contribution to the senescent phenotype is sparse [116,117]. Elevated ROS levels fuel lipid peroxidation and breakage of lipids, altering the permeability and fluidity of the membrane lipid bilayer and therefore cell integrity [11]. Increased availability of labile iron favors the generation of highly reactive lipid radicals (RO^•^) (see Figure 1B and Reaction (2)), which can drive detrimental cellular processes, including ferroptosis, an iron-dependent form of cell death [11]. RO^•^ (like HO^•^) are extremely reactive and have some very local effects. Moreover, the breakdown byproducts of lipid peroxidation, mostly reactive aldehydes such as malonaldehyde and 4-hydroxynonenal, are highly electrophile compounds, and they easily modify intracellular macromolecules (mainly proteins) by forming covalent electrophilic addition products. Such highly damaging lipid peroxidation products have been found in increased levels in cells and biological fluids in ageing and age-related diseases [59]. Concerning senescence, it has been reported that excess ROS generated from dysfunctional mitochondria induce lipid oxidative damage and lipid deposits [118,119]. Apart from lipid damage, products of lipid peroxidation are also elevated in senescent cells [117]. Finally, like proteins, oxidized lipids are also components of lipofuscin, as we will discuss below. 

### 4.4. Lipofuscin Formation and Accumulation 

#### 4.4.1. Lipofuscin: A Non-Degradable Product That Accumulates in Postmitotic and Senescent Cells

Lipofuscin is a pigmented, non-degradable biological “garbage” of intracellular catabolism, mainly found within lysosomes but also, in lesser amounts, in the cytosol [120,121]. It can be conventionally generated in virtually any cell type; however, its levels are nearly undetectable in normal proliferative cells, probably because its concentration is diluted with constant divisions. Still, lipofuscin progressively accumulates over time in postmitotic cells which no longer divide, and in cells undergoing senescence [1,121,122,123,124,125]. 

Despite its name, which implies a lipid structure (*lipos* is the Greek word for “fat”), lipofuscin is a heterogenous complex mixture consisting mainly of highly oxidized proteins, a lesser amount of lipids, and a few carbohydrates, ribonucleic acids, and metals [26,112], particularly iron ions [66]. The heavy cross-linking of the macromolecules within lipofuscin makes it difficult to identify its exact composition. However, there is strong evidence for the partial mitochondrial origin of lipofuscin [61,63,112,126]. 

An issue of debate is whether lipofuscin accumulation contributes to the initiation of senescence or whether it is just a mere consequence of this process. However, the finding that synthetic lipofuscin can by itself induce cellular senescence in human fibroblasts argues for a causal role in the process [127]. In any case, lipofuscin accumulation inhibits both proteasomal and lysosomal degradation systems, deteriorates cellular functionality, and is inversely correlated with longevity [128,129]. 

#### 4.4.2. Mechanisms of Lipofuscin Formation: The Role of Intracellular Iron Homeostasis and Oxidative Stress

Lipofuscin originates via oxidation and polymerization reactions of various cellular macromolecules and structures [26,121]. A requirement for the initiation of such devastating reactions is the generation of highly reactive oxidants. Intensive and/or prolonged oxidative stress leads to the formation of over-oxidized non-degradable materials [60,112] which finally aggregate, polymerize, and accumulate inside the cells, aggravating the rate of lipofuscin accumulation [61]. 

Lipofuscin resides primarily within lysosomes, acidic organelles with high-level degenerative potential that recycle non-essential, or eliminate harmful, cytoplasmic macromolecules and organelles delivered to them via the autophagy, phagocytosis, and endocytosis processes [130]. Since many of these materials contain iron, lysosomes are rich in this potentially harmful transition metal. The presence of labile iron and low pH make lysosomes ideal for Fenton-type reactions [131]. The generated highly reactive free radicals induce chain oxidation of lysosomal components leading to lipofuscin formation. This is supported by the observation that iron status regulates lipofuscin accumulation in rat heart myocytes cultured under oxidative stress conditions [132]. 

### 4.5. Alterations in Mitochondria 

Numerous studies highlight that senescent cells show remarkable alterations in mitochondrial function, structure, and dynamics [133]. In different models of cellular senescence, including oxidative stress-induced senescence, mitochondria become hyperfused and elongated, while their mass is increased [83,134,135,136,137]. Mitochondria are also less efficient in producing ATP and show increased proton leak and decreased mitochondrial membrane potential [83,134,138,139,140]. In addition, the autophagic degradation of dysfunctional or superfluous mitochondria within lysosomes (mitophagy) is impaired in senescent cells [141]. As a result, dysfunctional mitochondria which produce excessive ROS accumulate. It has been reported that mitochondrially derived ROS aggravate senescence by inducing genomic damage, particularly at telomeric regions, while interventions that diminish mitochondrial ROS hamper telomere shortening and extend the replicative lifespan [83,86,118]. Moreover, mitochondrial ROS are essential for the establishment of the senescent phenotype [83,134]. Particularly, excess ROS production from dysregulated mitochondria potentiates telomeric DNA damage and induces a prolonged DDR activation which is both necessary and sufficient for the establishment of stable cell growth arrest and senescence [134]. Mitochondrially derived ROS in senescence do not influence solely the genome but induce damage to other intracellular macromolecules as well. Recently, oxidatively modified proteins have been observed in the mitochondria of senescent cells [142], while ROS derived from dysfunctional mitochondria have been reported to induce lipid oxidative damage and lipid deposits [118,119]. Finally, mitochondrially derived ROS from senescent cells have been reported to spread senescence to neighboring healthy cells, through the induction of a DDR response [143,144]. 

Mitochondria contain high amounts of iron, up to 20–50% of the total cellular iron in some cell types [145]. Within the mitochondria, iron is primarily utilized for the biosynthesis of heme and Fe-S clusters; excess iron can be stored in the organelle-specific form of ferritin [33]. Iron accumulates in mitochondria with age, leading to mitochondrial dysfunction, increased oxidative damage, and cell death [146]. Similarly, mitochondrial iron overload correlates with oxidative stress and apoptotic cell death. However, evidence for the potential role of mitochondrial iron in senescence is sparse. It has been reported that frataxin deficiency, which is known to induce mitochondrial iron accumulation, provokes mitochondrial dysfunction, oxidative stress, and cellular senescence [147]. Moreover, a recent study in endothelial cells revealed that loss of neuropilin-1, a protein widely known for its role in angiogenesis, induces mitochondrial iron accumulation and iron-dependent oxidative stress, which results in mitochondrial dysfunction and senescence [148]. Additionally, treatment with mitoTEMO, a mitochondria-targeted antioxidant compound, or the iron-chelating drug deferoxamine (DFO), inhibited mitochondrial ROS production and protected against cellular senescence [148]. 

### 4.6. Alterations in Lysosomes 

Another common trait of senescent cells is alterations in lysosomal morphology and function. Lysosomes increase in number and size in senescence [4,8,63]. Moreover, as already mentioned, lysosomes contain high amounts of iron, which favors Fenton reactions [63,64,149] and makes these organelles ideal sites for the generation of lipofuscin t [128,131]. Lysosomal lipofuscin impairs autophagy and lysosomal degradation, further aggravating its formation and accumulation [128]. Moreover, lipofuscin seems to be another major intracellular source of ROS (together with mitochondria) in senescent cells, as the presence of iron within its structure catalyzes the formation of highly reactive free radicals [66,67]. Thus, lipofuscin itself can further oxidize cellular macromolecules, which has been reported to be a common trait of senescent cells [66]. Remarkably, as mentioned above, lysosomal dysfunction results in decreased mitophagy and the accumulation of damaged and dysfunctional mitochondria which produce elevated levels of ROS [63]. Mitochondrially derived ROS in senescent cells target intracellular macromolecules (DNA, protein, lipids) and organelles (including lysosomes), forming feedback loops that potentiate macromolecular and organelle damage and establish senescence [8,67]. 

## 5. Conclusions 

Cellular senescence is a cell state characterized by a generally irreversible cell cycle arrest [1]. This cell program plays beneficial roles in several physiological and pathological processes [2,5]. However, the prolonged accumulation of senescent cells within tissues and organs can be maladapted, contributing to a wide spectrum of age-related diseases [2,3,6]. Therefore, understanding the mechanisms that govern the initiation and establishment of cellular senescence, and ways to manipulate this process, is of major importance for biology and medicine. 

**Figure 4:**
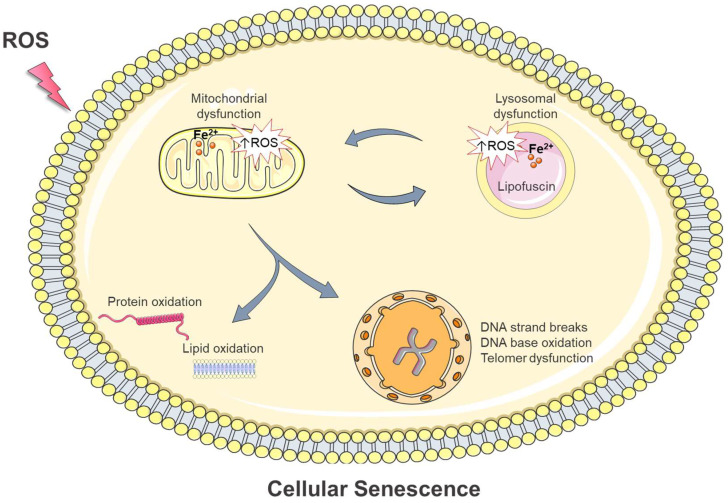
A summary of intracellular damage known to accumulate in cellular senescence caused by ROS. ROS induce oxidative damage to all essential macromolecules (DNA, protein, lipids) and organelle constitutes. Among organelles, mitochondria and lysosomes are more sensitive to oxidative stress conditions because of their high iron content. During senescence, lysosomes accumulate lipofuscin and display impaired autophagic degradation, diminishing mitochondrial turnover. Dysfunctional mitochondria accumulate and often produce excess ROS, further targeting intracellular macromolecules and lysosomes. These feedback loops potentiate cellular damage and aggravate cellular senescence. Iron most likely plays an essential role, as it catalyzes the generation of highly reactive free radicals able to induce oxidative damage to cellular constitutes. The figure was partly generated using Servier Medical Art, provided by Servier, licensed under a Creative Commons Attribution 3.0 unported license.

ROS are ultimately linked with cellular senescence. Elevated ROS trigger senescence by inducing macromolecular and organelle damage (Figure 4). Moreover, senescent cells —regardless of the initiation signal—often accumulate dysfunctional mitochondria which produce increased levels of ROS [63]. Mitochondrially-derived ROS are both necessary and sufficient for the establishment of a stable cell growth arrest and senescence [134].

ROS are free radicals or molecules produced by the incomplete reduction of O_2_. These species exhibit varying chemical reactivity and have pleiotropic effects on cells [10]. One-electron reduction of O_2_ generates O_2_^•−^, which is usually the first ROS to be formed. O_2_^•−^ is reduced into H_2_O_2_ by the action of SOD. Neither O_2_^−^ nor H_2_O_2_ is reactive enough to oxidize and induce damage to cellular macromolecules or organelle constitutes. Moreover, H_2_O_2_ plays an essential role as a physiological signaling agent. Yet, when labile iron is available, it can be converted via Fenton-type reactions into HO^•^, which is capable of oxidizing indiscriminately, and with high-rate constants, almost all cellular macromolecules [11,14]. 

Evidence in the literature is clear in highlighting that labile iron represents a requirement for ROS-induced adverse effects [11,14]. Moreover, we have previously demonstrated that the diminution of intracellular iron protects against oxidative stress-induced DNA damage and attenuates apoptotic cell death [101,102,103,104,105]. As such, the implication of iron in the mechanisms of oxidative stress-induced cellular senescence seems quite possible. In the literature, there is sparse evidence for the implication of iron in cellular senescence [106,150]. Recently, it was proposed that excess intracellular iron accelerates DNA damage via Fenton chemistry, and blocks genomic repair systems, causing cellular senescence [106]. Moreover, it was reported that iron levels regulate cellular senescence by altering p53 protein levels in mouse hepatocytes and rat liver [107]. In another recent study, the diminution of intracellular iron with the iron-chelating drug deferoxamine (DFO) prevented oxidative stress-induced cellular senescence of human-endometrium-derived mesenchymal stem cells (hMESCs) [151]. Evidence also exists for the implication of mitochondrial iron in oxidative stress-induced cellular senescence. It has been reported that the accumulation of iron within mitochondria induces iron-depended oxidative stress, resulting in mitochondrial dysfunction and cellular senescence [147,148]. Apart from mitochondria, lysosomes, as another iron-rich organelle [64,65], play a central role in oxidative stress and cellular senescence [8,67]. The presence of iron ions favors the generation of reactive free radicals, leading to the gradual accumulation of lipofuscin, which represents a universal hallmark of senescence [63,64,149]. Moreover, lipofuscin itself is a major source of intracellular oxidants, as it contains iron within its structure [66,67]. At the same time, the progressive deterioration of lysosomal function reduces mitochondrial turnover, resulting in increased mitochondrial ROS production in senescent cells [8,63,67]. Mitochondrially derived ROS, in turn, target intracellular macromolecules as well as lysosomes, forming feedback loops that potentiate cellular damage and aggravate cellular senescence [8,67]. 

On the other hand, there is evidence that iron accumulation is a consequence of cellular senescence regardless of the initiator stimuli (irradiative, replicative, or oncogenic) [152,153]. Masaldan et al. demonstrated that senescent cells display changes in the levels of iron homeostasis proteins, accompanied by the excessive accumulation of intracellular iron (up to 30-fold) [152]. Although the total level of intracellular iron increases, the senescent cells were remarkably highly resistant to ferroptosis, a type of iron-dependent cell death. This is probably because ferritin, the major intracellular iron-storage protein, also increased in senescent cells. Increased ferritin levels were driven by impaired lysosomal degradation of ferritin within lysosomes, a process known as ferritinophagy [152]. 

The aforementioned data strongly suggest that intracellular iron is involved in oxidative stress-induced senescence, yet its role is complicated, and further studies are required to unveil the precise roles of iron in this process. Targeting labile iron with iron-chelating drugs or diet-derived compounds with iron-chelating capacity has been shown to be an effective strategy to counteract the adverse effects of oxidative stress [101,102,103,104,105,154,155,156,157,158,159]. Thus, modulating labile iron might represent a hitherto unappreciated approach to prevent oxidative stress-induced cellular senescence.
